# Glycaemic and insulinaemic response to dietary fibre in horses

**DOI:** 10.1186/1751-0147-57-S1-P2

**Published:** 2015-09-25

**Authors:** Christine Brøkner, Dag Austbø, Jon Anders Næsset, Dominique Blache, Knud Erik Bach Knudsen, Anne-Helene Tauson

**Affiliations:** 1 Department of Veterinary Clinical and Animal Sciences, University of Copenhagen, Copenhagen, Denmark; 2 Department of Basic Sciences and Aquatic Medicine, Norwegian University of Life Sciences, Ås, Norway; 3 School of Animal Biology, University of Western Australia, Perth, Australia; 4 Department of Animal Science, Aarhus University, Aarhus, Denmark

## Introduction

Dietary sugar and starch affect plasma glucose and insulin concentration. Little information is available about the effect of dietary fibre on plasma glucose and insulin concentration. It is hypothesized that different dietary fibre compositions will alter post-prandial glycaemic and insulinaemic index of test diets.

## Objective

The objective was to measure postprandial glucose and insulin concentrations in horses fed diets of different fibre compositions.

## Material and methods

Four geldings in a Latin Square design were fed 4 diets: (H) timothy hay, (OB) whole oats and molassed sugar beet pulp (Betfor®), (BB) whole barley and Betfor®, chaff based concentrate (M). Starch did not exceed 1 g starch per kg BW per meal. The horses were fasted 8 hours prior to sampling. Blood was drawn via jugular vein puncture into heparinized vacutainer tubes at time 0 before the morning meal and again at time (min) 30, 45, 60, 75, 90, 105, 120, 135, 150, 165, 180, 210, 240, 270, 330, 390, 450, 510, 570 post feeding. Immediately after sampling, the tubes were centrifuged; plasma was harvested and stored at -20 °C. Blood plasma was analysed for insulin and glucose. Feeds were analysed for dietary fibre, non-starch polysaccharides, soluble non-cellulosic polysaccharides and NDF.

## Results

Mean area under the plasma curve (AUC) was 3101 (H), 3152 (OB), 3140 (BB) and 3275 (M) for glucose and 6670 (H), 9296 (OB), 8055 (BB) and 8334 (M) for insulin. The AUC did not differ significantly between diets.

## Conclusion

In conclusion, diets containing different fibre compositions did not affect the glycaemic and insulinaemic index in horses.

